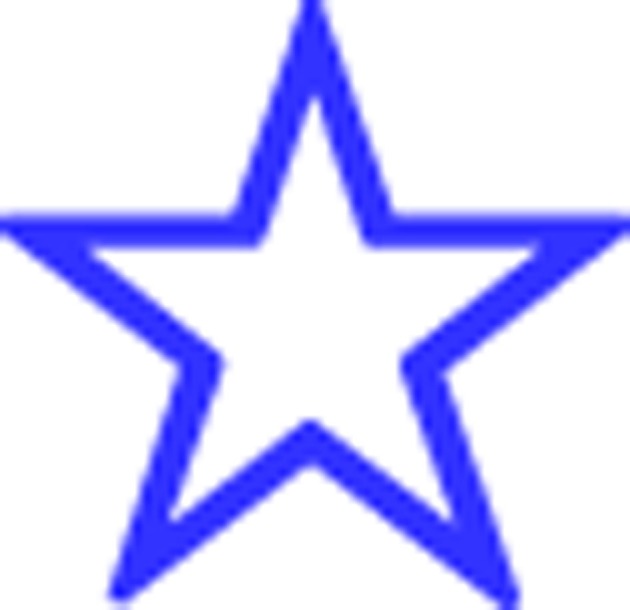# Improvement of system capacitance via weavable superelastic biscrolled yarn supercapacitors

**DOI:** 10.1038/ncomms13811

**Published:** 2016-12-15

**Authors:** Changsoon Choi, Kang Min Kim, Keon Jung Kim, Xavier Lepró, Geoffrey M. Spinks, Ray H. Baughman, Seon Jeong Kim

**Affiliations:** 1 Center for Self-Powered Actuation, Department of Biomedical Engineering, Hanyang University, Seoul 04763, Korea; 2 The Alan G. MacDiarmid NanoTech Institute, University of Texas at Dallas, Richardson, Texas 75083, USA; 3 Intelligent Polymer Research Institute, ARC Centre of Excellence for Electromaterials Science, University of Wollongong, Wollongong, NSW 2522, Australia

## Abstract

Yarn-based supercapacitors having improved performance are needed for existing and emerging wearable applications. Here, we report weavable carbon nanotube yarn supercapacitors having high performance because of high loadings of rapidly accessible charge storage particles (above 90 wt% MnO_2_). The yarn electrodes are made by a biscrolling process that traps host MnO_2_ nanoparticles within the galleries of helically scrolled carbon nanotube sheets, which provide strength and electrical conductivity. Despite the high loading of brittle metal oxide particles, the biscrolled solid-state yarn supercapacitors are flexible and can be made elastically stretchable (up to 30% strain) by over-twisting to produce yarn coiling. The maximum areal capacitance of the yarn electrodes were up to 100 times higher than for previously reported fibres or yarn supercapacitors. Similarly, the energy density of complete, solid-state supercapacitors made from biscrolled yarn electrodes with gel electrolyte coating were significantly higher than for previously reported fibre or yarn supercapacitors.

## Abstract (web summary)

Carbon nanotube yarns with high loadings of pseudocapacitive material are desirable, e.g., for emerging wearable technologies. Here authors make biscrolled yarns with high loadings of MnO_2_ nanoparticles confined in carbon nanotube galleries, demonstrating very high linear and areal capacitances.

Yarn and fibre-based supercapacitors are seen as a potential solution to the increasing energy demands provided by continued advances in wearable technologies. In addition to providing high-density energy storage and fast energy release, these fibres must also be sufficiently robust to be fabricated into textiles by such industrial processes as weaving, knitting and sewing. Important recent advances have increased the energy and power densities of fibre-based supercapacitors^1^,^2^,^3^,^4^,^5^,^6^,^7^,^8^,^9^,^10^,^11^,^12^,^13^,^14^,^15^,^16^,^17^,^18^,^19^,^20^,^21^,^22^,^23^,^24^,^25^,^26^,^27^,^28^,^29^,^30^, some of which also provide high flexibility and stretchability. Yet, the ever advancing applications needs far eclipse presently realized power and energy densities of yarns and fibres and new approaches are needed to bridge this technology gap.

Supercapacitors exploit electrochemical double-layer charge injection to provide the combination of high energy storage densities and fast charge and discharge. Performance is enhanced by balancing the competing demands for maximizing the interfacial area between active electrode material and electrolyte without compromising mechanical robustness or electronic conductivity. Pseudocapacitive active materials, such as manganese dioxide (MnO_2_), provide increased intrinsic capacitance, but need to be employed as fine powder or thin films because thick MnO_2_ layers can significantly degrade areal and volumetric capacitances and rate capability due to intrinsic low electrical conductivity^31^.

In this direction, recent improved fibre-based supercapacitors have used thin MnO_2_ coatings that are applied to highly conductive and, in some cases, highly stretchable base fibres^2^,^3^,^4^,^5^,^6^,^7^,^8^,^9^,^10^,^11^,^12^,^13^,^14^,^15^,^16^,^17^. Ultimately, however, this core–shell structure limits the allowable active material loading before performance is compromised. The fundamental problem is that only the shell is utilized as an effective loading site for active materials, while the bulk core of the fibre does not participate in the electrochemical charge/discharge processes^2^. Active material loadings have to date been restricted to <20 wt%, even when nano-structured core fibres have been used, such as twist-spun carbon nanotube (CNT) yarns^2^,^4^,^5^,^6^ and CNT-coated, coiled nylon fibre^3^.

To achieve both high discharge rate and high energy storage capabilities, we utilize a powerful technology called biscrolling^32^. This inexpensive method enables the continuous fabrication of electrically conducting yarns containing unspinnable particles by integrating these particles as a guest within the helical corridors in a twist-spun CNT yarn host. Biscrolling dramatically expands the achievable loading of active particles in yarns to as high as 99 wt%, and both the high loading levels and retention of useable strength has enabled the development of biscrolled yarn superconductors^33^, bio-fuel electrodes^34^ and battery electrodes^32^. Importantly for electrochemical applications, the active guest particles contained within biscrolled yarns retain their high surface areas and remain accessible by external electrolytes. Biscrolling MnO_2_ nanoparticles within a CNT host develops effective electronic and ionic conduction pathways through the continuous, helically wound CNT host and the layered, vasculature yarn corridors that secure the MnO_2_ nanoparticles in close contact with the electrolyte. Even with 90 wt% MnO_2_ loadings, the presently described biscrolled yarns remained flexible, stretchable, knottable and weavable. The biscrolled MnO_2_/CNT yarns have specific capacitances (*C*_A_=889 mF cm^−2^ and *C*_V_=155 F cm^−3^) and energy densities (*E*_A_=35.8 μW cm^−2^ and *E*_V_=5.41 mWh cm^−3^) that, to our knowledge, exceed the previously reported performances of fibre-based supercapacitor electrodes.

## Results

### Fabrication and morphology of biscrolled yarn supercapacitors

**Figure 1:**
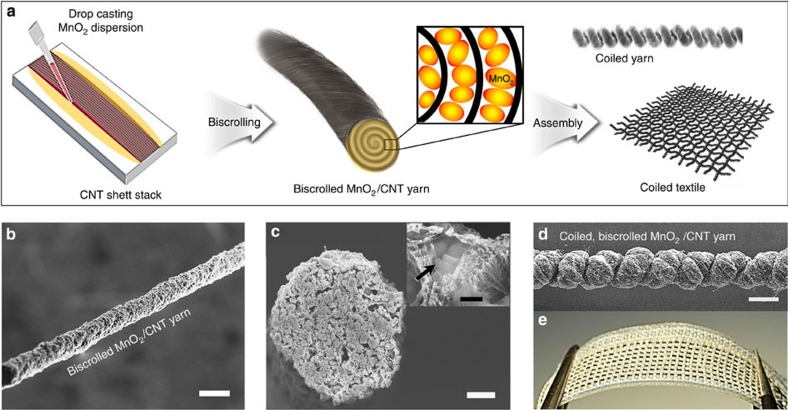
Fabrication scheme and images of biscrolled MnO_2_/CNT supercapacitor. (**a**) Schematic illustration of the fabrication of a biscrolled MnO_2_/CNT yarn electrode and a biscrolled yarn-based coiled and woven supercapacitor. SEM images of **b** a 91.1 wt% MnO_2_-loaded biscrolled yarn electrode (scale bar, 100 μm) and (**c**) its cross-section (scale bar, 15 μm) obtained by sectioning with a focused Ga ion beam. The inset is a high magnification image of a MnO_2_ particle (arrowed) surrounded by CNT bundles (scale bar, 500 nm). (**d**) SEM image of 5-ply, biscrolled 70 wt% MnO_2_/CNT yarn that was coiled by twist insertion (scale bar, 100 μm). (**e**) Photograph of eight 4-cm-long non-coiled, 91.1 wt% MnO_2_/CNT biscrolled yarn electrodes woven into a textile.

The fabrication of the biscrolled yarns aimed to maximize the loading of active MnO_2_ nanoparticles, while maintaining acceptable mechanical properties and electronic conductivity. The fabrication process started with the preparation of CNT aerogel sheet ribbon stacks as host material, which were drawn from a carbon multi-walled nanotube forest^35^. The MnO_2_ nanoparticles, which were dispersed by ultrasonication in ethanol, were subsequently drop-cast on the CNT sheet stack as shown in Fig. 1a and Supplementary Fig. 1. Ethanol acted as both dispersion medium for the MnO_2_ powder and densification agent for the CNT sheets. The MnO_2_-coated CNT sheets were then formed into biscrolled yarns by twist insertion to 2,000 turns per metre of initial sheet length. The loading level of guest MnO_2_ particle was controlled by altering the MnO_2_ concentration dispersed in the ethanol. The maximum loading level of MnO_2_ that could be successfully biscrolled was 93 wt%, which was produced using a ∼5 mg ml^−1^ concentration. Scanning electron microscope (SEM) images of the surface and cross-section of biscrolled 91.1 wt% MnO_2_/CNT yarn are shown Fig. 1b. At this high level of guest loading, the yarn surface was rough and internal pores were formed; however, the overall yarn was reasonably uniform in diameter and roughly circular in cross-section. Higher magnification imaging of the yarn cross-section (Fig. 1c) and energy dispersive spectroscopy and related elemental mapping confirmed the biscrolled structure (Supplementary Fig. 2), with partially aggregated MnO_2_ particles confined within the CNT scroll galleries on the inside of the yarn. The intimate contact between MnO_2_ aggregates and adjacent CNT bundles provides an interconnected network structure (Fig. 1c inset, Supplementary Fig. 3).

Highly stretchable supercapacitor yarns were constructed by overtwisting the biscrolled yarns to form coiled structures. In one example, five yarns having a high MnO_2_ content (70 wt%) were prepared and plied together as a 5-ply electrodes for additional strength and highly twisted (∼25,000 turns per metre) to form a stable, coiled structure (Fig. 1d). These coiled yarns contained 100 coils per centimetre of coiled yarn length and had a diameter of just ∼100 μm, which is less than one-third that of pervious stretchable supercapacitor fibre electrode based on CNT-coated nylon coiled fibre^3^. The coiled yarns showed spring-like stretchability with elastic strains to 30% demonstrated for the 5-ply coiled yarn with a MnO_2_ loading of 70 wt%. Coiling of biscrolled yarns with higher MnO_2_ loadings was not possible, because these biscrolled yarns broke during the coiling process. Despite the high concentration (up to 91.1 wt%) of brittle metal oxide powder contained within the biscrolled electrode, the yarns were mechanically strong and flexible enough to be hand-woven into a textile, as shown in Fig. 1e.

### Electrochemical performance of biscrolled supercapacitor

Symmetric electrochemical capacitors were prepared using either twisted or coiled biscrolled yarns as electrodes. Two such electrodes were arranged parallel and coated using an aqueous poly(vinyl alcohol) (PVA)/LiCl gel electrolyte to complete assembly of a solid-state yarn supercapacitor. For comparison, similar capacitors were prepared using neat twisted CNT yarns without any MnO_2_ guest.

**Figure 2:**
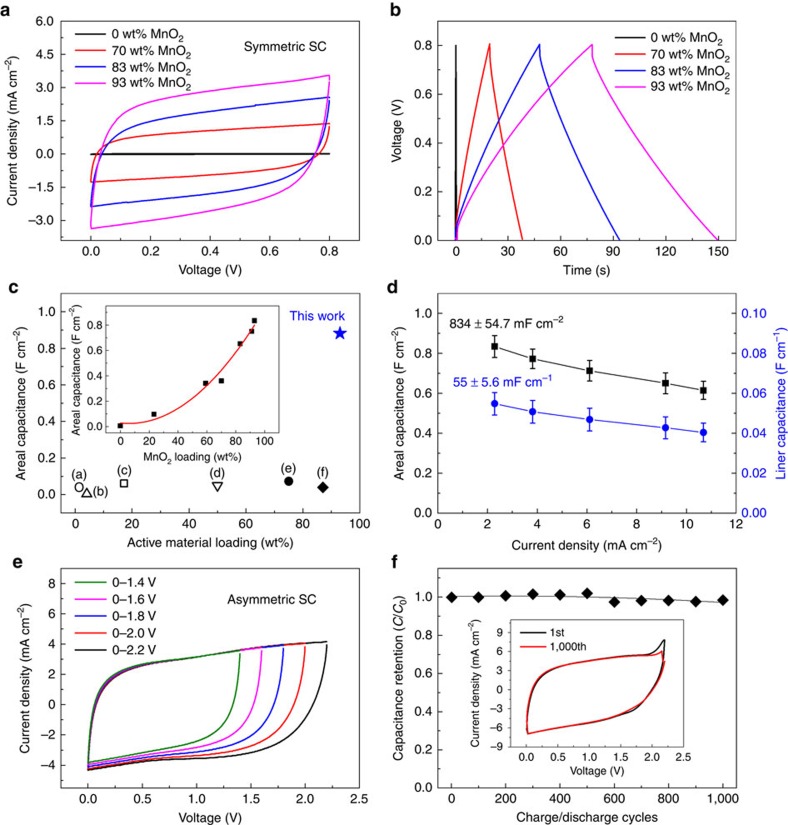
Electrochemical performance of biscrolled MnO_2_/CNT supercapacitors. (**a**) CV curves (at 10 mV s^−1^) and (**b**) galvanostatic charge/discharge curves of solid-state supercapacitors measured at 0.3 mA discharge current, which comprise two-symmetric biscrolled electrodes, as a function of MnO_2_ loading (0, 70, 83 and 93 wt%). (**c**) Single-electrode areal capacitances versus MnO_2_ loading level (wt%) of biscrolled supercapacitors (inset) compared with previously reported fibre supercapacitors using various amounts of active materials: (a) 1.45 wt% MnO_2_-coated CNT/nylon coiled fibres^3^, (b) 4.1 wt% MnO_2_-coated CNT yarn^5^, (c) 17.2 wt% MnO_2_-coated CNT coiled yarn^4^, (d) 50 wt% polyaniline coated CNT/rubber elastic fibre^14^, (e) 75 wt% poly(3,4-ethylenedioxythiophene) biscrolled CNT yarn^1^ and (f) 87 wt% ‘Ordered Mesoporous Carbon' nanocomposite fibre^18^. Inset: areal single-electrode capacitance versus MnO_2_ loading wt% for a current density of 2.3 mA cm^−2^. (**d**) Linear and areal single-electrode capacitances of solid-state supercapacitor, comprising two-symmetric, non-coiled, 93 wt% MnO_2_-loaded biscrolled yarns versus current densities from 2.3 to 10.8 mA cm^−2^ (error bars indicate±1 s.d. from four measurements). (**e**) CV curves with extended voltage range (at 100 mV s^−1^) of asymmetric supercapacitor, which comprises a 10-ply, non-coiled, RGO/CNT biscrolled anode and a non-coiled, MnO_2_/CNT biscrolled cathode. (**f**) Capacitance retention of the above asymmetric supercapacitor during charge/discharge cycles, showing 98.4% capacitance retention after 1,000 cycles. The inset compares CV curves before and after 1,000 charge/discharge cycles. All experiments in this figure were performed in a two-electrode system using PVA/LiCl gel as solid electrolyte.

Cyclic voltammetry (CV) curves (Fig. 2a) and galvanostatic charge/discharge curves for the capacitors made with neat CNT, and biscrolled electrodes with 70, 83 and 93 wt% MnO_2_ loading are compared in Fig. 2b. The box-like rectangular CV curves and triangular charge/discharge curves identify the absence of Faradic redox reactions and are consistent with energy storage by electrochemical double-layer charging capacitance of the CNT and the pseudocapacitance of MnO_2_ (ref. 3). From discharge peaks, about 46, 46 and 48 mV voltage drops were observed at discharge current density of 2.3 mA cm^−2^ for 93, 83 and 70 wt% biscrolled MnO_2_/CNT yarn supercapacitors, respectively (Supplementary Fig. 4). Areal capacitance values normalized to the total external surface area of a single electrode were calculated and found to dramatically increase with increase in MnO_2_ loading (inset of Fig. 2c). The highest values of linear- and area-normalized specific capacitances were 60.6 mF cm^−1^ and 889 mF cm^−2^, respectively, measured by galvanostatic charge/discharge curves using a discharge current of 2.3 mA cm^−2^. The areal capacitance of the neat CNT yarn was about 6 mF cm^−2^, which is comparable to values reported in the literature (1.97–8.66 mF cm^−2^)^5^,^18^,^24^. With incorporation of 93 wt% MnO_2_ loading, the areal capacitance dramatically increased to a peak value of 889 mF cm^−2^.

This capacitance enhancement is achieved by the unique internal structure formed by biscrolling, which maintains a high surface area of the MnO_2_ phase even at high loading levels. As illustrated in Fig. 2c, the areal capacitance of supercapacitors based on MnO_2_ and other pseudocapacitive materials presented in previous work is only moderately increased (by up to five times) by increasing the loading of active materials^6^,^8^,^9^,^10^. In contrast, biscrolled yarns with MnO_2_ contents of 60 wt% and above provided over 50 times higher areal capacitances than for the neat CNT yarn. Since the area occupied by a yarn in a textile increases in proportion to the external surface area of the yarn, these areal capacitances indicate the remarkable increase in the energy storage capability per textile area that can be realized by using biscrolled yarns. The kinetics of charging and the stability of the biscrolled yarn supercapacitors were also evaluated. As expected, the per-electrode capacitances of the all-solid-state biscrolled yarn supercapacitors decreased as the voltage scan rate increased. The neat CNT yarn retained 87% CV curve area, while the biscrolled 91.1 wt% MnO_2_ yarn retained only 31% as the scan rate increased from 10 to 100 mV s^−1^ (Supplementary Fig. 5). This degradation of rate capability can be explained by a decrease in yarn electrical conductivity from 170 to 29 S cm^−1^ in going from the neat yarn to a yarns with maximum MnO_2_ loading (Supplementary Fig. 6).

The linear and areal capacitances of biscrolled MnO_2_/CNT electrodes with 93 wt% MnO_2_ loadings (Fig. 2d) were determined from galvanostatic charge/discharge curves measured for current density from 2.3 to 10.8 mA cm^−2^ (Supplementary Fig. 7). The gravimetric capacitance of biscrolled 93 wt% MnO_2_/CNT yarn (based on total mass of electrochemically active materials, presently MnO_2_/CNT yarn) versus discharge current density are presented in Supplementary Fig. 8. The highest gravimetric capacitance is 166 F g^−1^ at current density of 2.3 mA cm^−2^. Self-discharge was characterized by measuring the open circuit voltage versus time for a charged MnO_2_/CNT biscrolled supercapacitor (Supplementary Fig. 9). A small voltage drop of 0.2 V was observed during self-discharge for 5,000 s. The stable voltage range for the symmetrical biscrolled yarn supercapacitors was as high as 1.2 V (Supplementary Fig. 10A), with only 8% loss in capacitance after 1,000 charge/discharge cycles (Supplementary Fig. 10B).

An asymmetrical capacitor was also fabricated to extend the working voltage range. Here the anode was constructed by biscrolling 18.5 wt% of chemically reduced graphene oxide (rGO) flakes as guest materials within the CNT host. The resulting CV curves of the asymmetric biscrolled supercapacitor comprising a 10-ply, biscrolled rGO/CNT yarn (anode) and a non-plied, biscrolled MnO_2_/CNT yarn (cathode) containing 91 wt% of MnO_2_ are shown in Fig. 2e. The working voltage was extended from 1.4 to 2.2 V without any redox peaks, and the asymmetric configuration also exhibited negligible loss in capacity after 1,000 repeated charge/discharge cycles (Fig. 2f). However, the asymmetric biscrolled supercapacitor shows lower capacitance (175 mF cm^−2^) than the symmetrical system because of the rGO-based anode's lower intrinsic charge storage capability.

### Stretchable and flexible biscrolled supercapacitor

**Figure 3:**
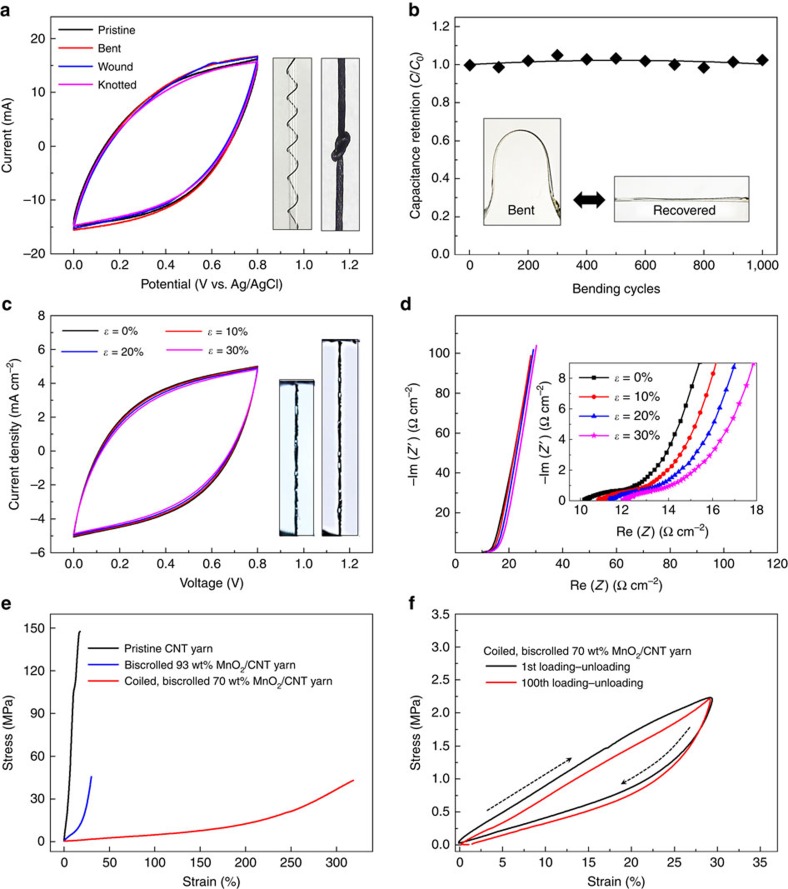
Performance of stretchable, bent, mandrel-wrapped and knotted MnO_2_/CNT biscrolled supercapacitors. (**a**) CV curves (at 50 mV s^−1^) for non-deformed, bent, mandrel-wrapped, and knotted biscrolled MnO_2_/CNT yarn. Insets: optical images showing a 173-micron-diameter biscrolled yarn electrode wound around a 1 mm diameter glass tube and a knotted 173-micron-diameter biscrolled yarn. These yarns contain 82 wt% MnO_2_. (**b**) Capacitance retention of the non-coiled, biscrolled electrode with 91 wt% MnO_2_ loading during repeat bending cycles. The inset shows optical images of bent (165° bending degree) and non-bent yarn on the supporting polyethylene terephthalate (PET) substrate used in the bending cycle evaluation. (**c**) CV curves (at 10 mV s^−1^) and (**d**) Nyquist curves measured for the initial (*ɛ*=0%) and statically stretched states (*ɛ*=10, 20, 30%) of the stretchable supercapacitors made from coiled, 5-ply, biscrolled 70 wt% MnO_2_/CNT yarn anodes and cathodes coated with PVA/LiCl gel electrolyte. The insets in **c** shows stretched (*ɛ*=30%) and released states of the coiled yarn supercapacitor and the inset in **d** shows higher resolution plots of the Nyquist curves for different applied strains. (**e**) Engineering stress–strain curves for pristine CNT yarn (black), biscrolled 93 wt% MnO_2_/CNT yarn (blue), and coiled, biscrolled 70 wt% MnO_2_/CNT yarn (red). (**f**) Engineering stress–strain curves for coiled, biscrolled 70 wt% MnO_2_/CNT yarn before (black) and after (red) 100 loading and unloading cycles.

One of the remarkable advantages of the biscrolled yarn electrodes is their high strength even with high loadings of brittle MnO_2_ nanoparticle guest. To evaluate flexibility, biscrolled 91 wt% MnO_2_/CNT yarns were bent to a maximum 165°, wound around a 1 mm diameter glass tube and even knotted. CV of the deformed capacitors fibres were almost indistinguishable from that for the non-deformed fibres (Fig. 3a). Even cyclic mechanical loading produced by repeated bending and straightening had little effect on supercapacitor performance. The electrochemical energy storage capacitance was fully maintained after 1,000 bending cycles from 0° to 165° (Fig. 3b).

Large tensile strains could also be accommodated without degrading electrochemical performance when biscrolled coiled yarns were used. Figure 3c shows optical images before and after 30% tensile strain was applied to a symmetrical solid-state supercapacitor fabricated from two coiled, 5-ply, biscrolled 70 wt% MnO_2_/CNT yarns and coated with PVA/LiCl gel electrolyte. Negligible changes were observed in the CV curves of the stretchable supercapacitor when strains of 10, 20 and 30% were applied. The inset photographs show the stretched and released states of coiled yarns, where high strain elasticity is achieved by reversible inter-coil separation. Electrochemical impedance spectroscopy conducted during coiled capacitor stretching (Fig. 3d) indicated a small increase in the initial equivalent series resistance from 10 Ω cm^2^ to 12 Ω cm^2^ after 30% tensile strain (inset of Fig. 3d). Unless otherwise mentioned, the electrodes are non-plied with 2,000 turns of twist if they are not coiled for flexible supercapacitor and five plied and contain 25,000 turns of twist if they are coiled for a stretchable supercapacitor. The engineering stress–strain curves for pristine CNT yarn; biscrolled 93 wt% MnO_2_/CNT yarn; and coiled, biscrolled 70 wt% MnO_2_/CNT yarn are shown in Fig. 3e. The tensile strength of the pristine yarn was 150 MPa and decreased to 45 MPa when 93 wt% MnO_2_ particles were incorporated by biscrolling. In addition, these results show that the strain-to-failure of the biscrolled yarn containing 70 wt% was very high (∼320%). The electrochemical performance for 93 wt% MnO_2_ particles biscrolled yarn supercapacitor was so highly retained against tensile deformation that about 99% CV area was conserved until yarn fracture (Supplementary Fig. 11).

To characterize elastic behaviour during cycling, stress–strain curves of the coiled, biscrolled 70 wt% MnO_2_/CNT yarn were obtained during 100 loading–unloading cycles, as shown in Fig. 3f. Although the coiled, biscrolled yarn showed hysteretic stress–strain curves and the degree of hysteresis decreased during cycling, no permanent deformation resulted from this cycling to 30% engineering strain. The mechanical energy loss of this coiled, biscrolled yarn was 43% during the first load–unload cycle and decreased to 41.6% for the 100th load–unload cycle.

### Specific capacitances and energy and power densities

**Figure 4:**
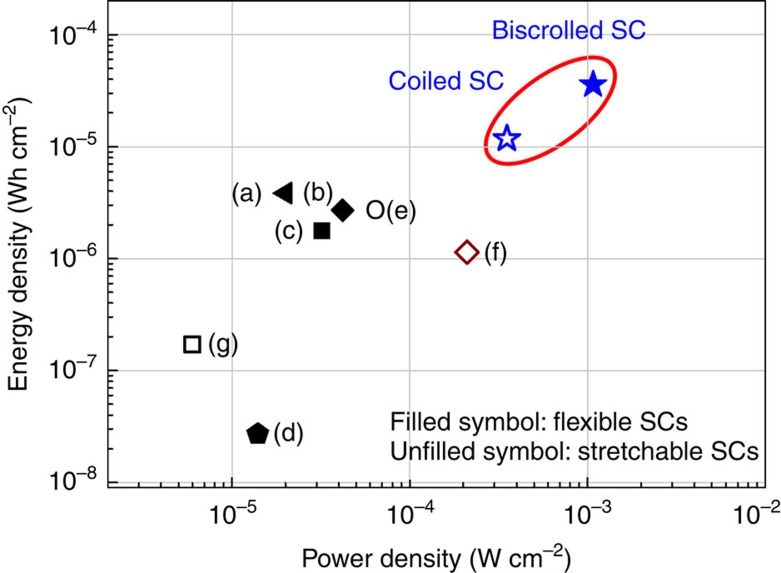
Energy density and power density of biscrolled MnO_2_/CNT supercapacitor compared with published results. A Ragone plot of areal energy density versus areal power density for biscrolled MnO_2_/CNT yarn supercapacitors, based on the total surface area of the complete supercapacitor, including biscrolled yarn and PVA/LiCl based gel electrolyte. Previously published data of yarn or fibre supercapacitors are included for comparison. Flexible, non-elastic fibre supercapacitors are denoted using filled symbols, while flexible, elastomeric supercapacitors are denoted using unfilled symbols. The maximum measured areal energy densities of presently investigated flexible non-coiled, biscrolled 93 wt% MnO_2_/CNT supercapacitor (filled blue star) and coiled 5-ply, biscrolled 70 wt% MnO_2_/CNT stretchable supercapacitor (unfilled blue star) are 35.8 and 11.7 μWh cm^−2^, respectively. The energy density of our non-coiled, flexible supercapacitor is higher than previous flexible supercapacitors, which comprise (a) CNT/graphene wet-spun fibres (3.84 μWh cm^−2^)^22^, (b) pen ink wires (2.7 μWh cm^−2^)^16^, (c) flexible ordered mesoporous carbon/CNT yarns (1.77 μWh cm^−2^)^18^ and (d) MnO_2_/Kevlar fibres (0.027 μWh cm^−2^)^8^. The energy density of the stretchable coiled supercapacitor is also higher than for previous stretchable supercapacitors comprising (e) MnO_2_/CNT/nylon coil fibres (2.6 μWh cm^−2^)^3^, (f) graphene spring fibres (1.14 μWh cm^−2^)^7^ and (g) MnO_2_/CNT spring fibres (0.17 μWh cm^−2^)^9^.

A Ragone plot (Fig. 4) was constructed to map the areal energy and power densities obtained from charge/discharge testing to 1.2 V (Supplementary Fig. 10A). These were calculated on the basis of the total surface area of the supercapacitor, including gel electrolyte coating. The maximum measured energy densities were 35.8 and 12 μWh cm^−2^ for the presently investigated flexible and stretchable supercapacitors made from, respectively, biscrolled 93 wt% MnO_2_/CNT and coiled, biscrolled 70 wt% MnO_2_/CNT electrodes. The energy density of our flexible biscrolled supercapacitor is higher than previously described flexible supercapacitors, such as those fabricated from CNT/graphene wet-spun fibres (3.84 μWh cm^−2^)^22^, PEN ink/wires (2.7 μWh cm^−2^)^16^, flexible OMC/CNT wires (1.77 μWh cm^−2^)^18^ and MnO_2_/Kevlar fibres (0.027 μWh cm^−2^)^8^. Similarly, the energy density of the stretchable coiled supercapacitor is higher than for any previous stretchable supercapacitors, including those based on MnO_2_/CNT/nylon coil fibres (2.6 μWh cm^−2^)^3^, graphene spring fibres (1.14 μWh cm^−2^)^7^ and MnO_2_/CNT spring fibres (0.17 μWh cm^−2^)^9^. Moreover, the maximum linear and volumetric energy densities of the biscrolled 93 wt% MnO_2_/CNT supercapacitor were higher (*E*_*L*_=5.91 μWh cm^−1^, *E_V_*=5.41 mWh cm^−3^) than for CNT fibre-based capacitor wire (*E_L_*=0.085 μWh cm^−1^)^18^, all carbon coaxial fibre supercapacitors (*E*_*L*_=0.7 μWh cm^−1^, *E*_*V*_=0.14 mWh cm^−3^)^12^ and wire shaped asymmetric supercapacitors (*E*_*L*_=0.043 μWh cm^−1^, *E*_*V*_=1.44 mWh cm^−3^)^7^. In addition, the average areal power density of flexible (using 93 wt% MnO_2_/CNT electrodes) and stretchable (using coiled, 5-ply, biscrolled 70 wt% MnO_2_/CNT electrodes) supercapacitors were 1,080 and 350 μW cm^−2^, respectively. Linear, areal and volumetric capacitances of present flexible supercapacitor (using biscrolled 93 wt% MnO_2_/CNT electrodes) and stretchable supercapacitor (with coiled, 5-ply, biscrolled 70 wt% MnO_2_/CNT electrodes) are shown and compared with other flexible/stretchable fibre supercapacitors in Table 1. The highest specific capacitances (denoted as *C_L_*, *C_A_*, and *C_V_*) for the flexible biscrolled supercapacitor were 60.6 mF cm^−1^, 888.7 mF cm^−2^ and 154.7 F cm^−3^, respectively, when measured at a current density of 2.3 mA cm^−2^ and based on the dimensions of a single electrode. These capacitances are higher than that reported for yarn or fibre-based supercapacitors despite the host of pseudocapacitive materials used^1^,^5^,^6^,^8^,^17^,^18^,^22^,^24^,^25^,^30^. Similarly, the present coil-based stretchable supercapacitors (*C_L_*=17.7 mF cm^−1^, *C_A_*=382 mF cm^−2^, *C_V_*=105 F cm^−3^) provide specific capacitances that far exceed equivalent values from previously reported stretchable fibre-based supercapacitors^3^,^4^,^7^,^9^,^14^,^26^,^29^.

## Discussion

The present combination of high power and high energy storage capabilities lies in the novel inner structure of the biscrolled yarn; the scroll structure provides a continuous pathway for both ion and electron from surface to centre, as well as sufficient porosity through scroll layers^1^. In addition, the resulting biscrolled yarn exhibited a complex network structure where MnO_2_ nanoparticles confined with CNT scroll galleries provide a high electrochemically active surface area. Since we have deployed the universal twist-insertion-based biscrolling and coiling method to obtain high performances and elasticity for fibre supercapacitors, the presently described method can be extended to diverse host and guest materials, such as polymer-based electrospun mats and other pseudocapacitive materials, respectively.

## Methods

### Preparation of biscrolled yarn electrodes

MWCNT sheets were drawn from a CNT forest (∼400 μm high and consisting ∼12 nm diameter nanotubes containing ∼9 walls), which was fabricated by a chemical vapour deposition method^33^. Commercially available MnO_2_ nanoparticles (rod shape with 30 nm diameter and 100 nm length, Sigma-Aldrich) were dispersed in ethanol (1∼5 mg ml^−1^) and ultrasonicated (1 h with 150 W, VCX 750). After drop casting the prepared MnO_2_ dispersion (∼100 μl cm^−2^) on the CNT sheets, the MnO_2_/CNT sheets were twisted to ∼2,000 turns per metre using an electrical motor to make the flexible biscrolled yarn electrode. To fabricate the stretchable electrode, five biscrolled 70 wt% MnO_2_/CNT yarns were placed in parallel and additionally twisted to a total of ∼25,000 turns per metre. This level of twist insertion was sufficient to fully coil the plied yarn. One end of each electrode was connected to a 180 μm diameter Cu wire using silver paste for electrochemical performance measurements.

### Supercapacitor assembly

The PVA/LiCl gel electrolyte was prepared by heating a mixture of 3 g PVA (*Mw* 146,000∼186,000) and 6 g LiCl in 30 ml deionized water at 90 °C for several hours. Two yarn electrodes were placed parallel and ∼100 μm apart and then coated with PVA/LiCl gel electrolyte to complete fabrication of the yarn supercapacitor. The woven textile supercapacitors were made by weaving biscrolled MnO_2_/CNT yarns into a textile using a needle and then coated with PVA/LiCl gel electrolyte. All chemicals for electrolyte synthesis were purchased from Sigma-Aldrich.

### Characterization

Electrochemical measurements on complete biscrolled yarn supercapacitors utilized a two-electrode configuration and an electrochemical analyzer (CHI 627b, CH Instrument). For cross-section analysis, the 91 wt% MnO_2_/CNT biscrolled yarn was cut using a focused Ga ion beam (FIB, Nova 200) operated at 30 kV. The clean-cut yarns were transferred to a Zeiss Supra 40 SEM to perform the microscopy and elemental mapping analysis. The length and weight of the yarn electrodes were measured using a digital Vernier caliper (500 series, Mitutoyo) and micro-balance (XP6, Meter toledo), respectively. The diameter for the yarns was measured by counting pixels of optical images taken using a microscope (Zoom 70XL Lens, Samsung). Mechanical properties were measured by thermal mechanical analyzer (TMA, SS7100) with strain of 0.85% per minute.

### Calculation of the electrochemical performances

The capacitance for two-electrode system was calculated from galvanostatic charge/discharge curves. From *C*=*I*/(d*V*/d*t*), where *I* is the discharge current and the d*V*/d*t* is the slope of discharge curve, the single-electrode specific areal capacitance (*C_sa_*) was calculated from following equation.


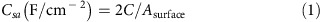


Where *A*_surface_ is the total external surface area of a single MnO_2_/CNT biscrolled yarn electrode. Total length and volume of the biscrolled yarn were used for linear and volumetric capacitance calculations, respectively. For a given constant scan rate *ν* and initial discharge voltage (*V*_i_), the average power was calculated by integrating the current (*I*) versus voltage (*V*) curves^1^;


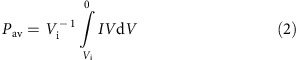


The energy was calculated by using equation (3);


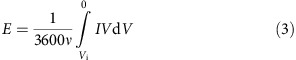


Resulting areal energy and average power densities indicated in Fig. 4 are for the complete supercapacitor (normalized by the total surface area of the supercapacitor which is taken as two times the external surface area of an electrolyte-coated MnO_2_/CNT biscrolled electrode).

### Data availability

The data that support the findings of this study are available from the corresponding author upon request.

## Additional information

**How to cite this article:** Choi, C. *et al*. Improvement of system capacitance via weavable superelastic biscrolled yarn supercapacitors. *Nat. Commun.* **7,** 13811 doi: 10.1038/ncomms13811 (2016).

**Publisher's note:** Springer Nature remains neutral with regard to jurisdictional claims in published maps and institutional affiliations.

## Supplementary Material

Supplementary Information Supplementary Figures.

## Figures and Tables

**Table 1:**
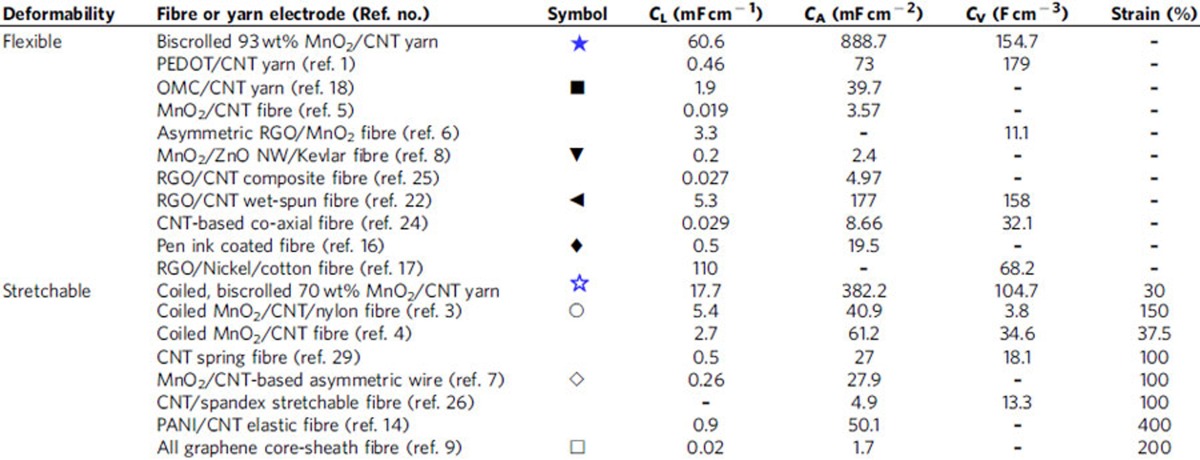
Comparison of specific capacitances for present and prior-art yarn or fibre supercapacitors.

**Figure:**
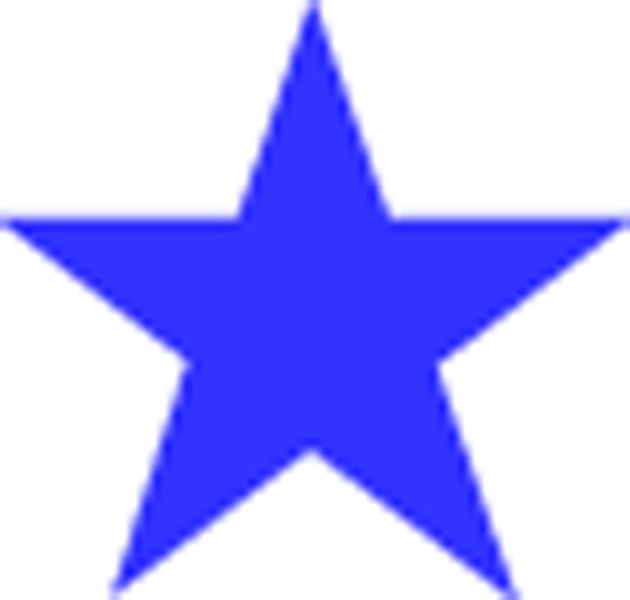


**Figure:**