# Supplementary material for: Improvement of system capacitance via weavable superelastic biscrolled yarn supercapacitors
Source: Nat Commun. 2016 Dec 15;7:13811. doi: 10.1038/ncomms13811 (PMC5172384; doi:10.1038/ncomms13811)
Supplement: Supplementary Information — Supplementary Figures. [file ncomms13811-s1.pdf]

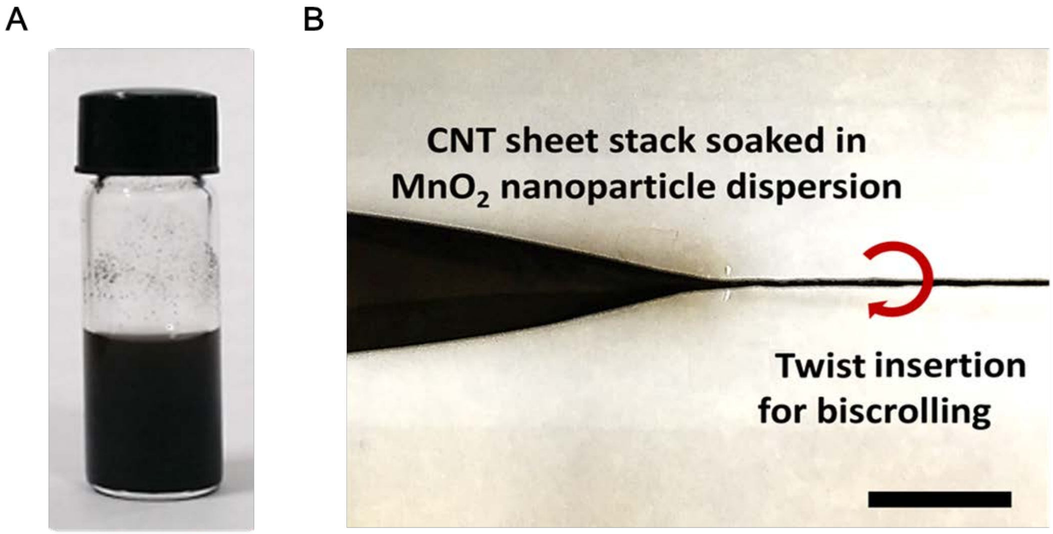

Supplementary Figure 1. Photographs of **A)** MnO_2 nanoparticle/alcohol dispersion (5 mg/ml) and **B)** the biscrolling process by drop-casting the MnO_2 dispersion on the CNT sheet stacks and twisting the MnO_2/CNT composite sheets about 2,000 turns/meter using an electrical motor (scale bar = 5 mm).

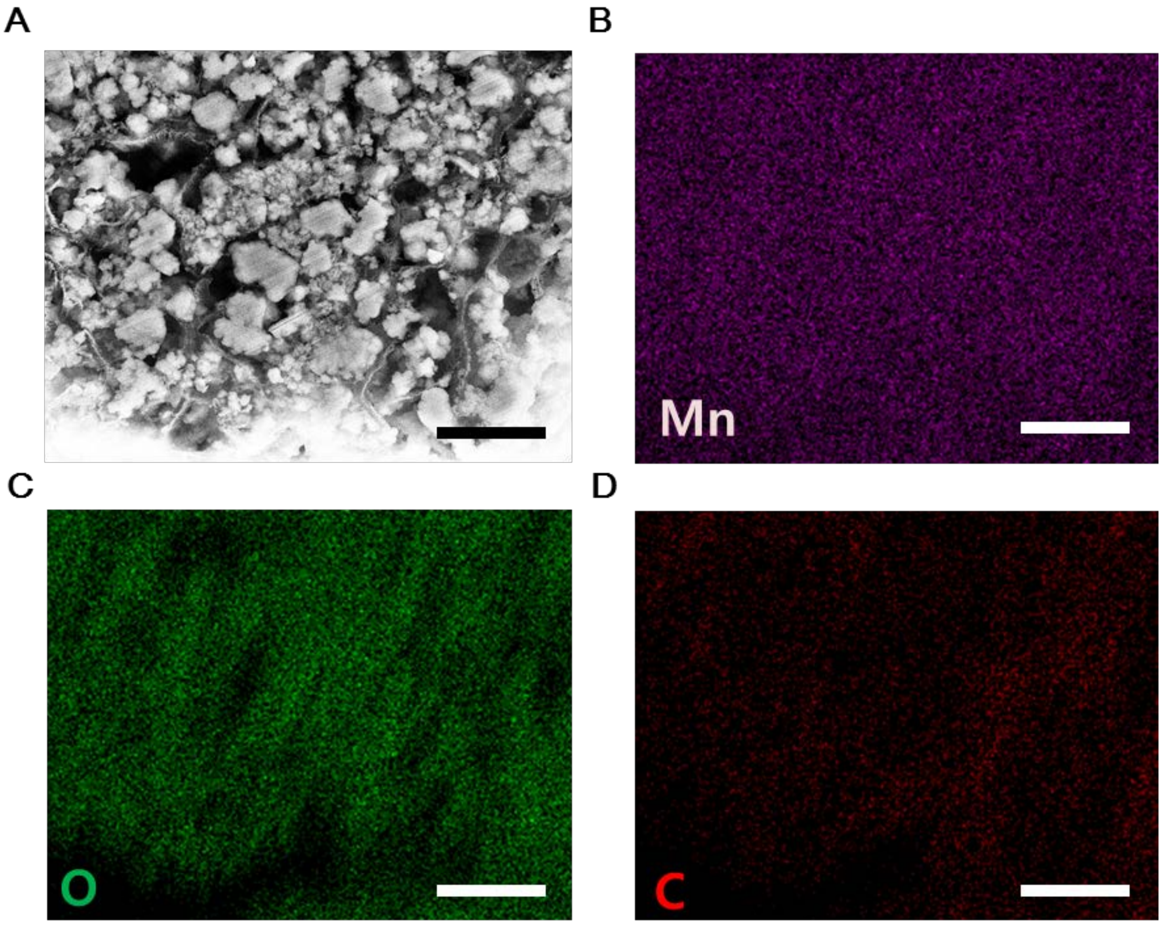

Supplementary Figure 2. SEM image **A)** and energy dispersive x-ray (EDX) element mapping results for the cross section of a biscrolled non-plied, 91 wt% MnO_2/CNT non-coiled fiber, where **B), C)**, and **D)** show manganese (purple dots), oxygen (green dots), carbon (red dots). All scale bars are 5 μm.

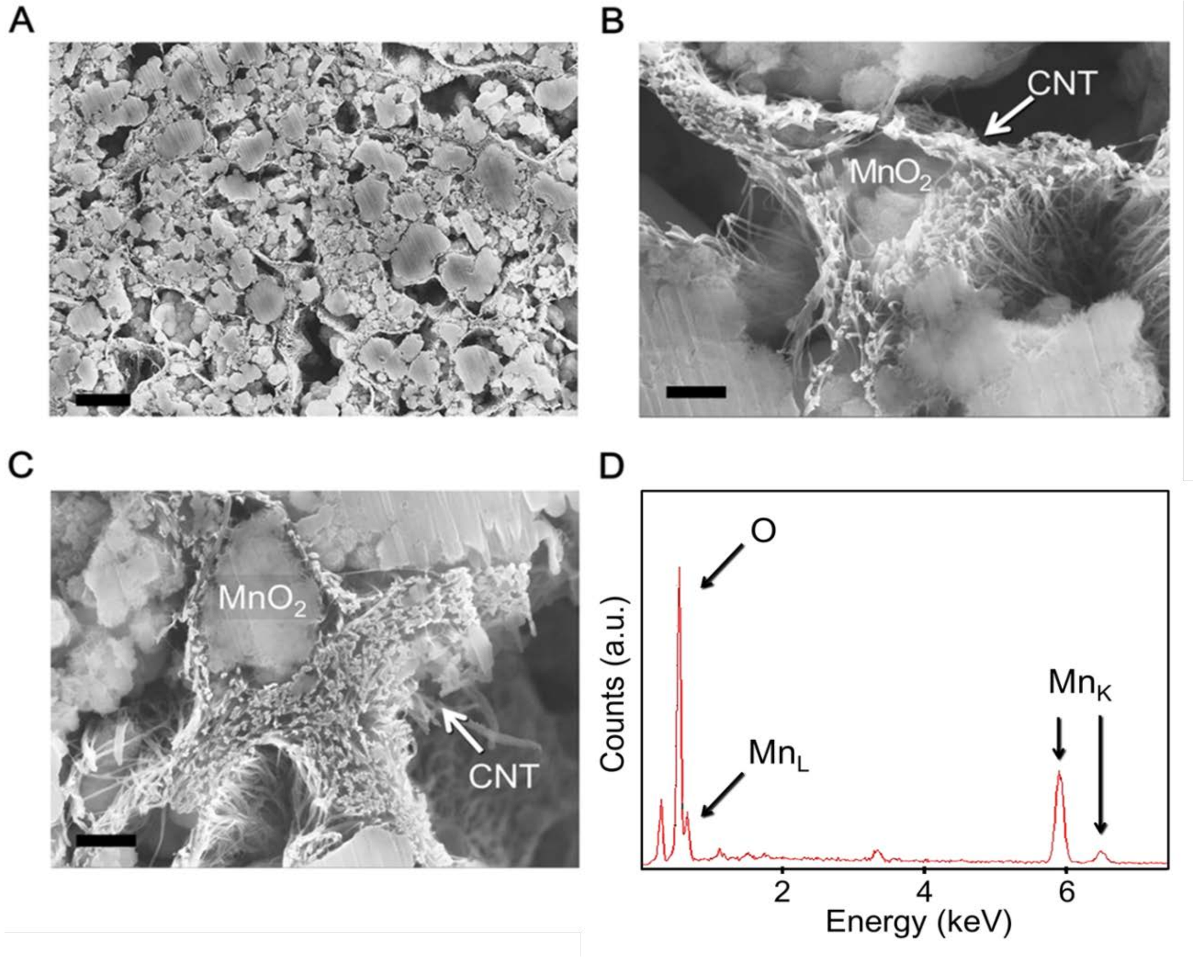

Supplementary Figure 3. A) Cross section of a non-coiled, 91wt% MnO_2/CNT biscrolled fiber obtained by cutting the fiber with a focused Ga ion beam (scale bar = 5 μm). **B)** and **C)** are magnified images showing MnO_2 nanoparticles confined by CNTs, where the scale bar in B and C is 700 nm. **D)** EDX analysis of the yarn cross section, which shows the intensity of Mn_L, Mn_K, and O peaks.

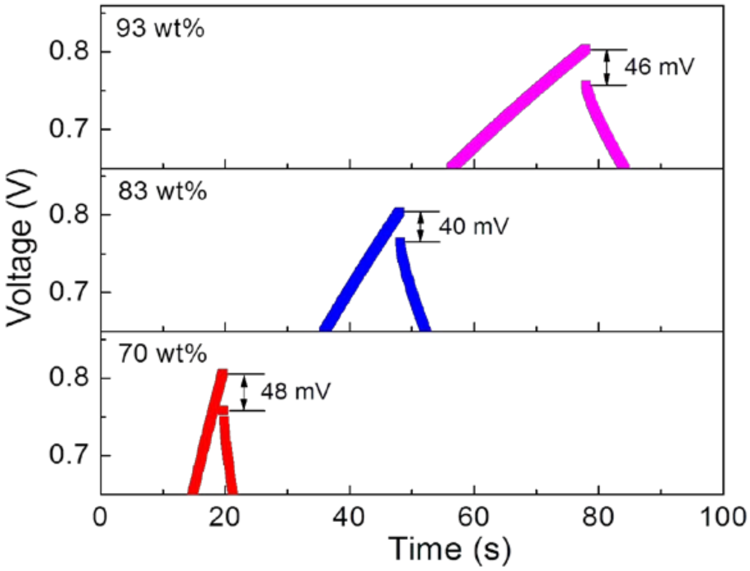

Supplementary Figure 4. Voltage drop for 93, 83, and 70 wt% biscrolled MnO_2/CNT yarn supercapacitors during discharge at current density of 2.3 mA/cm^2.

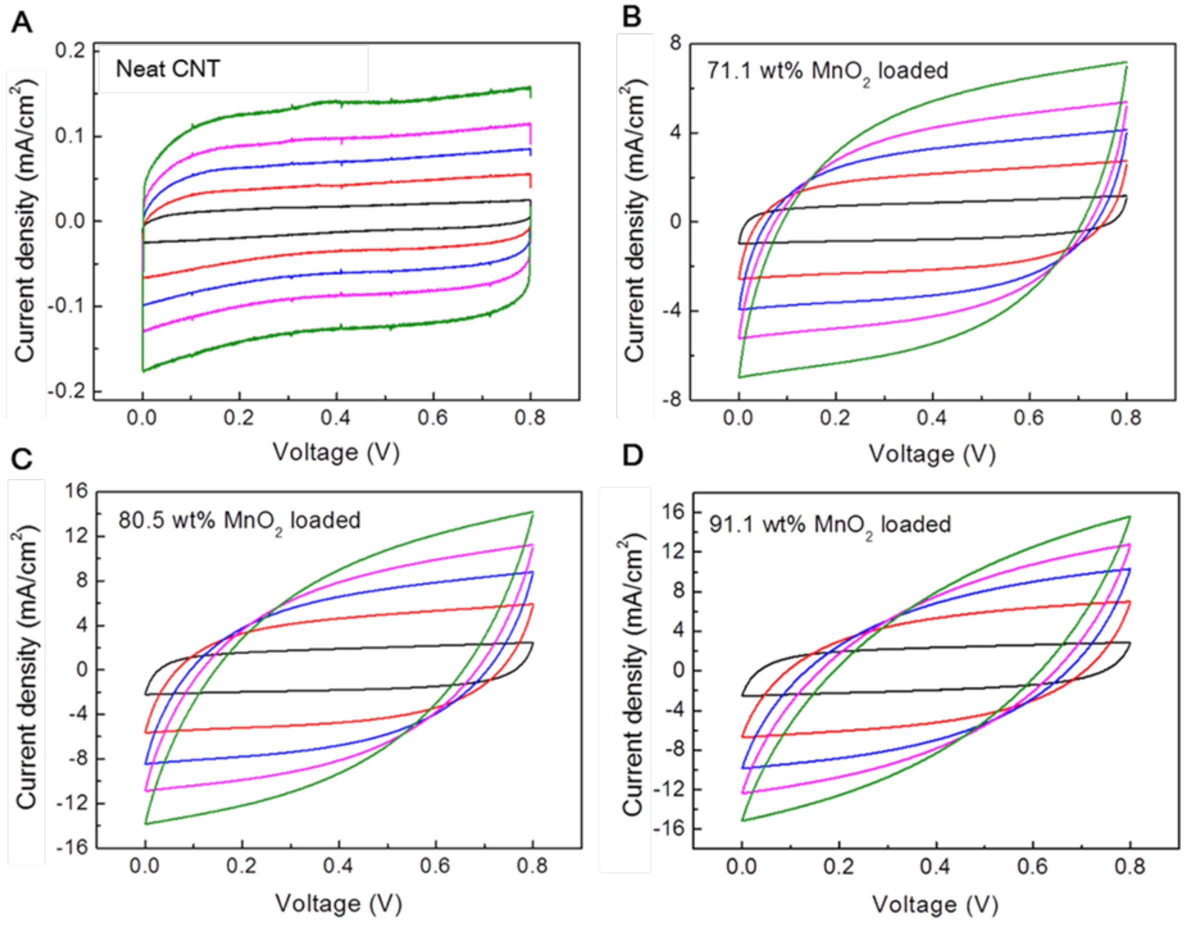

Supplementary Figure 5. CV curves (measured at scan rates from 10 to 100 mV/s in two electrode system) of solid-state supercapacitors comprising symmetric biscrolled electrodes with **A)** 0 wt%, **B)** 71.1 wt%, **C)** 80.5 wt%, and **D)** 91.1 wt% MnO_2 loadings.

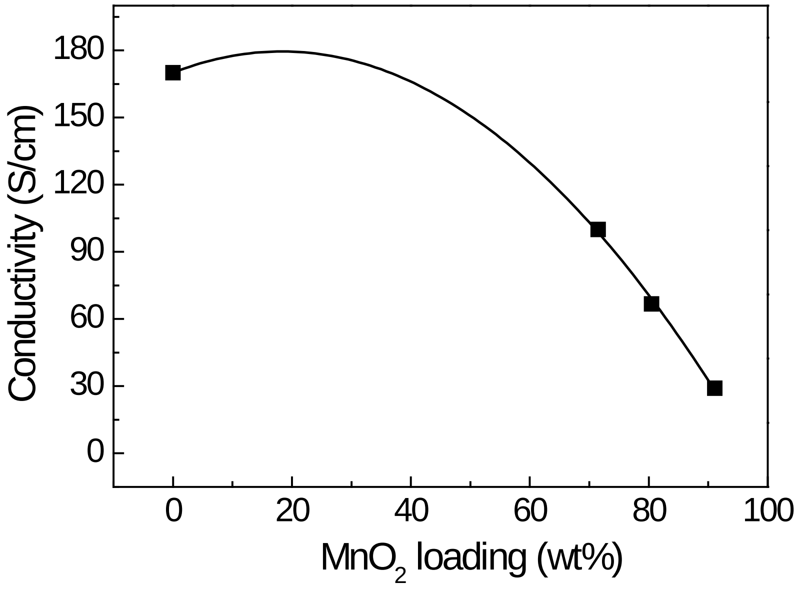

Supplementary Figure 6. The electrical conductivity of non-coiled, MnO_2/CNT biscrolled electrode versus loading level of MnO_2. The solid curve is a guide for the eyes.

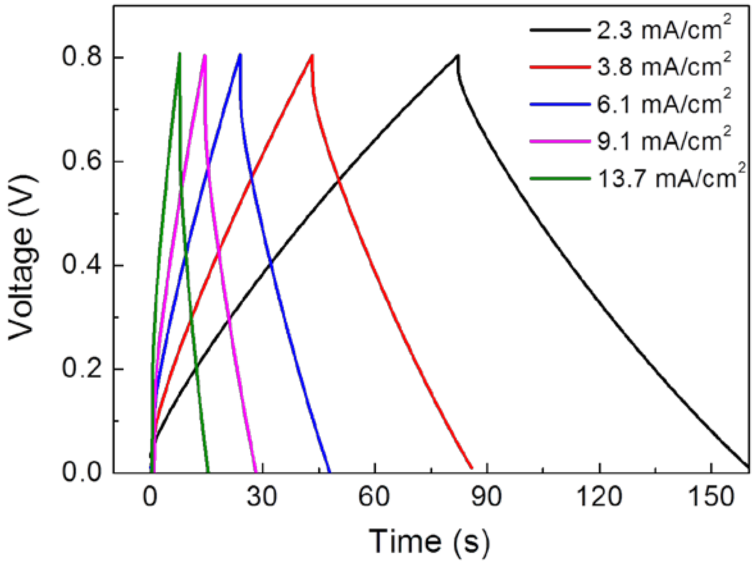

Supplementary Figure 7. The galvanostatic charge/discharge curves of biscrolled 93 wt% MnO_2/CNT yarn for current densities of from 2.3 to 13.7 mA/cm^2.

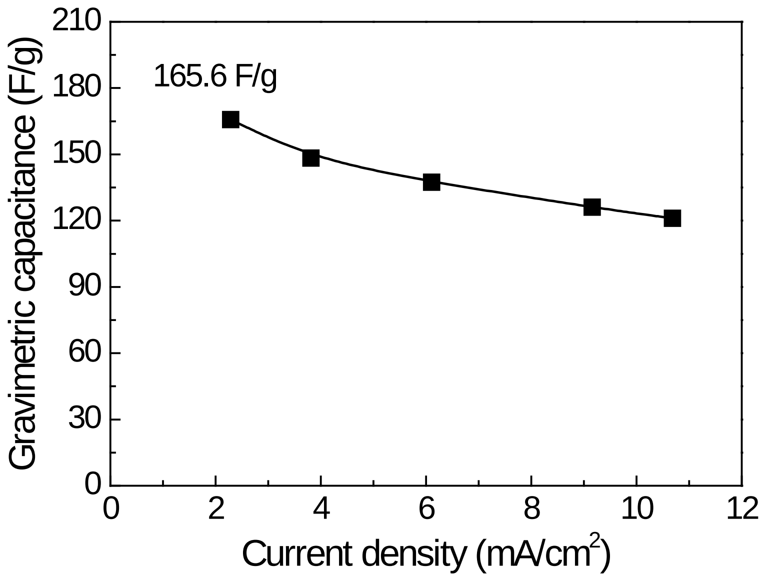

Supplementary Figure 8. Galvanostatic charge/discharge curves of a biscrolled 93 wt% MnO_2/CNT yarn for current densities of from 2.3 to 13.7 mA/cm^2.

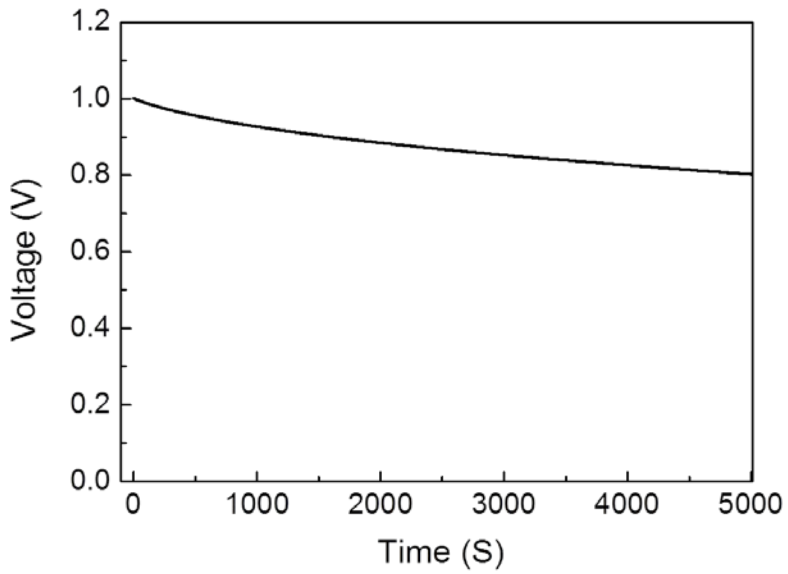

Supplementary Figure 9. Self-discharge curve showing open circuit voltage versus time for a solid-state supercapacitor comprising two symmetric, non-coiled, 91 wt% biscrolled MnO_2/CNT biscrolled fibers coated with a PVA/LiCl gel electrolyte.

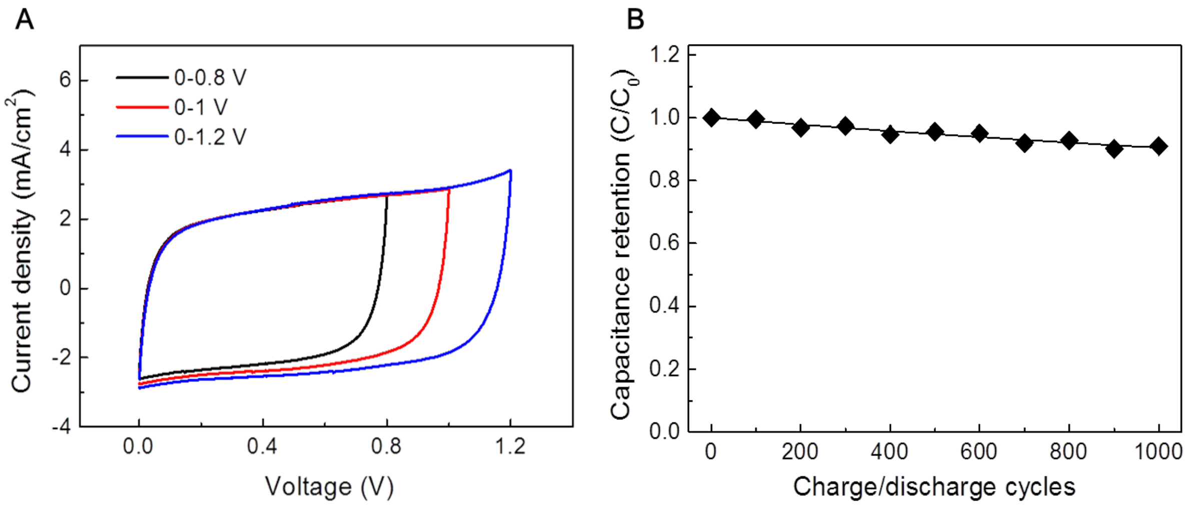

Supplementary Figure 10. **A)** CV curves measured using different working voltage ranges and a scan rate of 10 mV/s. **B)** Capacitance retention versus number of charge/discharge cycles performed by repeated CV measurements over a voltage range 0-1.2 V. All measurements are performed in two-electrode system of symmetric, non-coiled, 91 wt% biscrolled MnO_2/CNT yarn supercapacitor coated with a PVA/LiCl gel electrolyte.

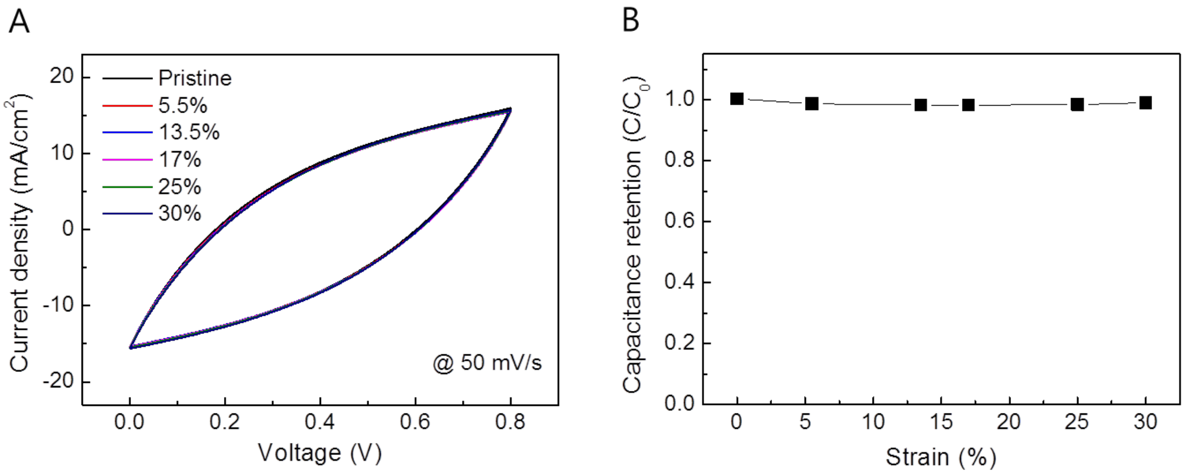

Supplementary Figure 11. **A)** CV curves for symmetric, solid-state, 90wt% MnO_2 biscrolled yarn supercapacitor compared at different tensile deformation. **B)** Capacitance retention versus applied tensile strain.
